# Erin Go Bragh

**Published:** 1900-03-24

**Authors:** 


### "Erin Go Bragh."

Even in the most strictly scientific or professional or commercial journal, during the last few months of national anxiety and suspense, it has been almost as impossible for any writer to keep the war out of his article as it was for the immortal Mr. Dick to exclude the head of Charles the First from the famous and never-ending memorial. The war has been with us wherever we went, and in whatever duty or business we were engaged. Superficially regarded, it would not be difficult to think of it altogether as an evil; and yet we know, as Von Moltke justly wrote many years before his fame had ripened, that "peace is a dream, and not even a beautiful dream." Nations wake from it to find their youth renewed, their energies redoubled, their fortitude increased, their minds diverted from mere luxury and ease, or from the considerations which the Apostle summed up in the single word "covetousness," to fresh perceptions of the dignity of courage and endurance, of the beauty of self-sacrifice, of the deathlessness of fame, of the rewards of conscience. The weakest among so-called philanthropists, and the silliest of doctrinaires, have always decried the soldier, and have expended their trivial vocabularies in his dispraise; only to see that the world, heedless of their cackle, has estimated him at his true value as soon as the necessity for his activity has arisen from the progress of events. Among other soldiers, when the time for the due appreciation of their labours has arrived, the soldier surgeon has occupied a prominent place, and has been conspicuous for the single-mindedness and courage with which he has trodden the path of duty, even when it has led only to the goal of death. From the time of Ambroise Paré downwards, through Guthrie and his fellow labourers in the Peninsula, to MacCormac, and Treves, Stokes, and Thomson, the needs of warfare have served to force upon the attention of the ruling classes of the country alike the surpassing value of surgical assistance and the spirit in which it has invariably been rendered, and has therefore served to increase the estimation in which the surgical profession has been held by those who, in the last resort, chiefly dominate and determine public opinion. The surgeon, in time of war, has opportunities greatly more numerous and more extended than those which fall to him in time of peace; and he is enabled to earn a reputation which survives the conditions in which it originated.

Among the many advantages likely to accrue to the British Empire from the war in which we are engaged not the least is the very manifest *rapprochement* which it has already produced between Great Britain and Ireland. It has long been the sincere desire of every Englishman to look upon every Irishman just as a man from Middlesex would look upon a man from Yorkshire or Devon; that is to say, as a brother Englishman, coming from a locality which has local associations and traditions of which he is justly proud, and from which he would refuse to detach himself, but which leave him none the less a loyal subject of the Empire, rejoicing in its prosperity and its glory, and eager to contribute towards their increase. Notwithstanding this, it must be confessed that many Englishmen had become tired of seeing their Irish fellow subjects dance to the wires of the political agitator, and have viewed with an approach to impatience the antics of such a body as the "Nationalist" members, or the Dublin Corporation. These minor irritations have already to some extent disappeared beneath the wave of admiration which has been felt, all over the world, for the bravery of the Irish soldiers of the Queen, and for the headship of the gallant Field Marshal, who has opened to them the road to victory. From the Queen herself, or from the Prince and Princess of Wales, to the humblest Englishman who on St. Patrick's Day displayed his sprig of shamrock in acknowledgment of Irish skill and courage, there has been evidence of a renewal of kindly feelings which had been partially concealed beneath rivalries or animosities artificially produced, and incapable, let us hope, of retaining their vitality in a more healthy moral atmosphere. It behoves Englishmen to do what may be in their power to maintain this atmosphere; and there is a possible step in this direction which would, we think, commend itself to the medical profession. A large proportion of our soldier surgeons are Irishmen, and have been second to none of the fighting line in their absolute devotion to duty. The Fellows of the Royal College of Surgeons of Ireland have a grievance of long standing, insomuch that, in spite of the privileges conferred by the Medical Act of 1858, they do not stand on the same plane with Fellows of the English College in respect of eligibility for certain hospital appointments. Is it not time that this grievance should be removed? It is one which could hardly survive a strong expression of opinion from the authorities in Lincoln's Inn Fields; and such an expression the present President would be an eminently proper person to initiate.

